# Polyester-Based (Bio)degradable Polymers as Environmentally Friendly Materials for Sustainable Development

**DOI:** 10.3390/ijms16010564

**Published:** 2014-12-29

**Authors:** Joanna Rydz, Wanda Sikorska, Mariya Kyulavska, Darinka Christova

**Affiliations:** 1 Bulgarian Academy of Sciences, Institute of Polymers, Acad. Georgi Bonchev St., Bl. 103A, Sofia 1113, Bulgaria; E-Mails: mkyulavska@polymer.bas.bg (M.K.); dchristo@polymer.bas.bg (D.C.); 2 Polish Academy of Sciences, Centre of Polymer and Carbon Materials, 34 M. Curie-Sklodowska St., Zabrze 41-800, Poland; E-Mail: wsikorska@cmpw-pan.edu.pl

**Keywords:** microbial and synthetic polyester, polylactide, polyamide, poly(ester amide), renewable resource

## Abstract

This review focuses on the polyesters such as polylactide and polyhydroxyalkonoates, as well as polyamides produced from renewable resources, which are currently among the most promising (bio)degradable polymers. Synthetic pathways, favourable properties and utilisation (most important applications) of these attractive polymer families are outlined. Environmental impact and in particular (bio)degradation of aliphatic polyesters, polyamides and related copolymer structures are described in view of the potential applications in various fields.

## 1. Introduction

Polymeric materials currently play an important role in everyday life due to their unique range of properties. Compared to other traditional materials, plastics offer many advantages, such as performance and versatility, durability, lightness, resilience to corrosion, ease of processing, high productivity, low cost, environmental aspect, *etc.*, which determine their importance to society and enables even greater extent of industrial development in the coming years. Innovation in plastics will thus make a valuable contribution to increasing economic growth and quality of life and help to solve environmental problems. Now, there is a remarkable increase in the potential use of biodegradable polymers in various areas such as medicine, pharmacy, agrochemistry or the packaging industry. This fast growing scientific area relates directly to the most crucial health and social problems nowadays [1]. Introduction of new environmentally friendly and sustainable plastics in the packaging and end-user industries is the solution to major problems in waste management. This can be achieved through information dissemination and by identifying and removing the barriers to faster and more widespread use of sustainable plastics, particularly biodegradable and those based on renewable resources. Ecological concerns have resulted in an increase of interest in renewable resources-based products. In the perspective of sustainable development, biodegradable polymers can be considered as safe for the environment, and are an interesting alternative to conventional polymers [2,3]. A number of biodegradable polymers have been developed in recent decades. With regard to the origin of the raw material they are divided into two groups: (i) biodegradable polymers from renewable resources (e.g., polymers of microbiological origin as well as synthetic polymers from renewable monomers); and (ii) biodegradable polymers of non-renewable/fossil resources. [4]. However, it is important to underline that aliphatic polyesters synthesised either by polycondensation of hydroxy acids or diacids and diols or by polymerisation of lactone-type heterocyles are not biopolymers [5]. Aliphatic polyesters play a predominant role as (bio)degradable polymers due to the potentially hydrolysable ester bonds and relatively short aliphatic chains present in the macromolecules and are the most representative examples of environmentally relevant polymeric materials [5,6]. On the other hand, they often lack good mechanical and physical properties, which can be compensated by development of copolymer structures such as poly(ester amide)s. In this review, aliphatic polyesters (in particular, polyhydroxyalkanoates and polylactides) as well as polyamides are summarised as the main representatives and most promising (bio)degradable polymers and their favourable properties, (bio)degradation and applications in various fields are described. The purpose of this review was not only to highlight the importance of polymers from renewable resources, such as polyhydroxyalkanoates, but also to emphasise their synthetic analogues such as isotactic, atactic and syndiotactic poly(3-hydroxybutyrate). Moreover, the possible preparation pathways and valuable properties of new copolymers, blends and composites based on polyhydroxyalkanoates from renewable resources containing synthetic poly(3-hydroxybutyrate) have not been covered broadly in the published surveys.

## 2. Main Routes of Polyesters Degradation

Generally, polymer degradation takes place mostly through scission of the main chains or side-chains of macromolecules, induced by thermal activation, oxidation, photolysis, radiolysis, or hydrolysis [7]. Polymer hydrolytic degradation may be defined as scission of chemical bonds in the polymer backbone, by water uptake, to form oligomers and finally monomers. In the first step, water molecules attack the water-labile bonds by either direct access to the polymer surface or by imbibition into the polymer matrix followed by bond hydrolysis [8]. In addition to nucleophilic attack by H_2_O (neutral hydrolysis), the hydrolysis can be also catalysed with an acid, base or enzyme.

### 2.1. Alkali-Catalysed Polyester Hydrolysis

**Figure 1:**
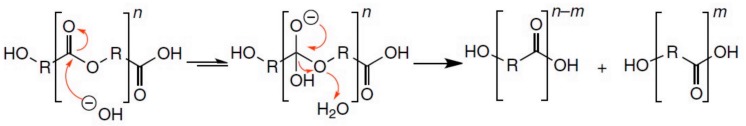
Alkaline-catalysed hydrolysis of polyesters, originally published in [9].

The first step of degradation under alkaline conditions is the attack of the hydroxide anion to the carbonyl carbon of the ester group, generating a tetrahedral intermediate. This step is reversible and the hydroxyl attached to the tetrahedral intermediate can leave, resulting in the regeneration of the ester. However, the ether connected to the tetrahedral intermediate (RO^−^) can also leave, resulting in hydrolysis, *i.e.*, generation of an alcohol and carboxylic acid. The preference of the tetrahedral intermediate toward hydrolysis *versus* ester regeneration is determined by the ability of the leaving alcohol (ROH) to stabilise a negative charge; therefore, esters formed from acidic alcohols hydrolyse faster than ester formed from aliphatic alcohols (Figure 1) [9]. As a result, one hydroxyl and one carboxyl end group is generated [10].

Higher degradation rates in alkaline medium were observed compared with those at acidic conditions [10,11,12].

### 2.2. Acid-Catalysed Polyester Hydrolysis

**Figure 2:**
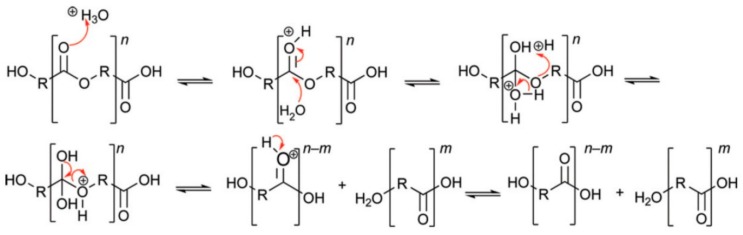
Acid-catalysed hydrolysis of polyesters, originally published in [9].

Under acidic conductions degradation of polyesters begins with protonation of the carbonyl oxygen of the ester group by a hydronium ion, which makes the carbonyl carbon more electrophilic due to the positive charge. This is followed by attack of water molecules on the carbonyl carbon, which generates a tetrahedral intermediate similar to the one generated during base-catalysed hydrolysis. The tetrahedral intermediate can then decompose into a carboxylic acid and an alcohol, or regenerate the original ester (Figure 2) [9]. In the acid hydrolysis, protonation of the chain oxygen atom of the ester group, followed by reaction with water produces one hydroxyl and one carboxyl end group [10].

### 2.3. Enzymatic Degradation of Polyesters

**Figure 3:**
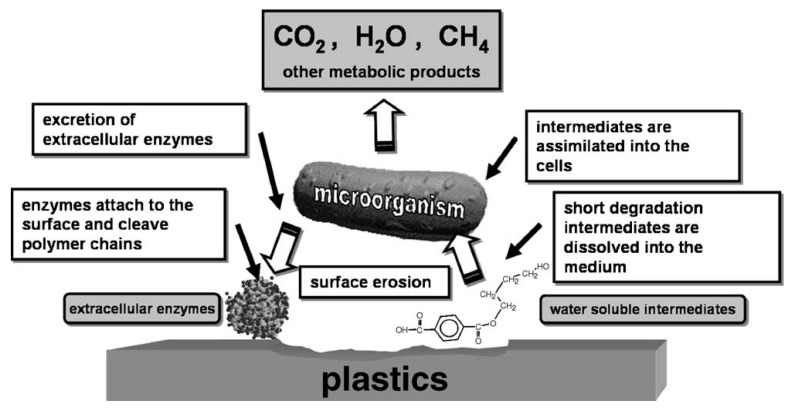
Scheme of general mechanism of enzymatic catalysed hydrolytic polymer degradation, originally published in [14].

Some polymers undergo degradation in biological environment when microorganisms are present around the polymer materials. Such environment includes soil, sea, river and lake as well as body of human being and animal [7]. Biodegradation of polymers is the attack of microorganisms on predominantly water insoluble polymer-based materials [13]. Due to water insolubility and high-molar mass of the polymer molecules, microorganisms are not able to incorporate the polymers, by outer cell membranes, directly into the cells where most of the biochemical processes take place. Therefore, excretion of extracellular enzymes occurs first which depolymerise the polymers outside the cells (Figure 3) [14]. When the molar mass of the polymer is sufficiently reduced to water-soluble intermediates, they can be introduced into the microorganisms’ metabolic pathways. As a final result of these processes end products such as water, carbon dioxide, methane (in the case of anaerobic degradation), and new biomass are produced [14,15].

## 3. Polyhydroxyalkanoates—Polyesters of Microbiological Origin and Their Synthetic Analogues

Natural polyhydroxyalkanoates (PHA)s are polymers from renewable resources which mean that the final polymer is directly converted from such non-fossil resource [16]. They are widely distributed in biological systems and attract increasing attention in many fields such as medicine, surgery, pharmacology, agriculture, packaging industry, biotechnology, polymer waste management, *etc.* [6,17]. Synthetic PHAs analogues offer greater advantages over natural polymers since they can be tailored to give a wider range of properties and to obtain tailor-made biodegradable material for specific applications in different areas [6,18,19].

### 3.1. Poly(3-hydroxyalkanoate)s from Renewable Resources—Synthesis and Properties

**Figure 4:**
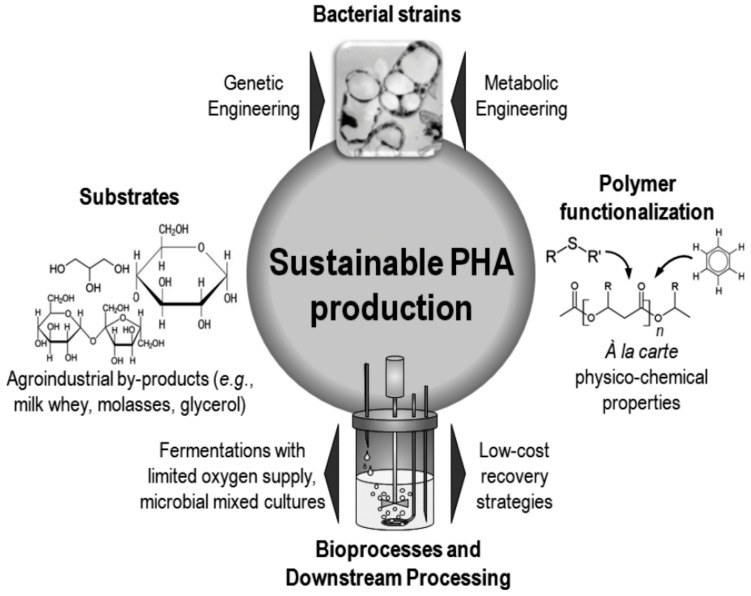
Strategies used to enhance the sustainability of polyhydroxyalkanoates (PHA) production processes, originally published in [21] under CC BY 3.0 license.

Poly(3-hydroxyalkanoate)s belong to a family of fully biodegradable, thermoplastic polyesters formed by several kinds of bacteria as carbon and energy reserves. They are produced not only by bacterial fermentation [20,21] (Figure 4), but also by transgenic microorganisms [22] and plants [23].

Plant cells can produce only a small quantity of PHA. In bacteria, PHAs are accumulated at 90% of the dry cell mass. Bacterial fermentation of PHAs uses sugar and fatty acids as carbon and energy sources [24]. This agricultural feeds are processed directly by the enzymatic activity to the polymer under balanced growth conditions when the cells become limited for an essential nutrient but are exposed to an excess of carbon [16,25].

Poly[(*R*)-3-hydroxybutyrate] was the first representative of PHAs described by Lemoigne in 1920s. Three types of natural poly[(*R*)-3-hydroxybutyrate] (PHB) with different numbers of 3-hydroxybutyric acid units (HB) and different functions have been identified in living organisms: (i) high-molar-mass storage PHB in cytosolic inclusion bodies of various microorganisms, consisting of 10,000 to >1,000,000 HB residues; (ii) low-molar-mass PHB in cell membranes (complexed with polyphosphates) consisting of 100–300 residues; and (iii) short-chain oligo[(*R*,*S*)-3-hydroxybutyrate] (OHB) (≤30 HB units) covalently conjugated to proteins, found even in human tissues [25,26,27,28].

The physical properties of PHAs vary from crystalline-brittle to soft-sticky materials depending on the length of the side aliphatic chain on β-carbon [29,30]. However, the high crystallinity of PHB limits their wide applications, especially as packaging. The melting point (*T_m_*) ranges from 173–180 °C and the glass transition temperature (*T_g_*) is around 5 °C [6]. The polymer is too brittle for most practical applications and materials obtained therefrom possess poor mechanical properties. To reduce the crystallinity and improve the properties, PHB can be copolymerised with the structural segments derived from selected natural PHAs. Copolymerisation of PHB with 5%–20% 3-hydroxyvaleric acid units (HV) allows the production of PHBV copolymer with improved mechanical properties and such material can be used as biodegradable packaging [31]. Functional PHAs can be prepared via biosynthetic procedures by incorporating various functional monomers or other metabolites in the polymer sequence. For example, fermentation of *Alcaligenes eutrophus* in a culture containing polyethylene glycol or polysaccharide has given a hydroxy-terminated block copolymer consisting of PHA [32,33].

### 3.2. Synthetic Poly(3-hydroxybutyrate)s—Polymers from Fossil Resources

Synthetic analogues of the natural PHB, with potential industrial importance are obtainable by direct copolymerisation of epoxides with carbon monoxide and via ring-opening polymerisation (ROP) of β-butyrolactone to isotactic, atactic and syndiotactic poly(3-hydroxybutyrate) [34,35,36]. The common anionic initiators activated by the addition of macrocyclic ligands such as crown ethers or cryptandes or by using bulky counter-ions or suitable highly polar aprotic solvents as e.g., DMSO are able to initiate polymerisation of β-butyrolactone [37,38,39,40]. The polymer chain growth proceeds regioselectively and stereoselectively entirely *via* carboxylate anions. Propagation on carboxylate active centres (much less sensitive to impurities than any other anionic species) enables the scaling up the anionic ring-opening polymerisation process up to industrial level. Short-chain PHB can be obtained from high-molar-mass PHB by degradation according to E1cB elimination mechanism which leads to oligomers with crotonate end groups or by thermal degradation [41,42,43,44,45]. The results of the fundamental research on the synthesis of homo- and copolymers have been patented [46,47].

The purity of β-butyrolactone used in the polymerisation process affects the molar mass of the obtained polymer. Synthesis of high-molar-mass PHB by anionic polymerisation must be carried out in a solvent-free system [39], and requires strict control of the reaction temperature and extraordinary care in the purification of the monomer. Regioselective anionic ring-opening polymerisation of β-lactone is accompanied by side reactions leading to the formation of polymers with unsaturated end groups. Unsaturated (crotonic) end groups during polymerisation of β-butyrolactone may be formed both in the initiation and propagation process. During isothermal degradation of synthetic atactic PHB (aPHB) with carboxylate end groups, a significant decrease of the molar mass and formation of oligomers with crotonic end groups as degradation products has been observed, in contrast to aPHB terminated by carboxy end groups. Therefore, it is very important to control the temperature during the polymerisation in order to avoid side reactions leading to a reduction of the polymer molar mass in relation to the assumed value. The recognition of this phenomenon allows for the synthesis of aPHB with a molar mass of more than 100,000 g/mol and its use as a component in poly(lactide-*co*-glycolide) (PLGA) compositions for the preparation of bioresorbable (degradable with proved elimination from the human body) fibres. This convenient and solvent-free method can also be used for the controlled degradation of natural PHA under mild conditions towards the preparation of slower degrading oligomers of predetermined molar mass and defined end groups. [48]

### 3.3. Copolymers, Blends and Composites Based on Microbial and/or Synthetic Poly(3-hydroxyalkanoate)s

Tailoring of the polymer architecture of biodegradable and biocompatible polymers and their synthetic analogues is the way to improve the properties and develop novel biodegradable materials [49]. PHA of natural origin can be modified to produce tailored block, graft and random copolymers containing structural segments derived from synthetic analogues and other synthetic polymers such as polycaprolactone (PCL), poly(methyl methacrylate) (PMMA), poly(lactic-*co*-glycolic acid) or synthetic PHB resulting in various materials with special features. Moreover, PHB graft copolymers were prepared by various polymerisation methods, for example, anionic polymerisation of β-butyrolactone on poly(methyl methacrylate) multifunctional macroinitiator to synthesise P(MMA-*graft*-aPHB) containing synthetic poly[(*R*,*S*)-3-hydroxybutyrate] side chains [50]. Atactic or isotactic PHB macromonomers functionalised with methacrylate end groups were copolymerised in a one-step procedure with several methacrylates via grafting through by atom transfer radical polymerisation (ATRP) [51]. Recently, a water-soluble brush copolymers composed of synthetic PHB and poly(ethylene glycol) (PEG) brushes were prepared applying a three-step procedure, including ATRP processes [52]. Such copolymers exhibit a wide range of interesting properties, with potential application in many fields especially in medicine and environmental protection. More recently, the controlled synthesis of PHB-PEG-PHB triblock copolymer by β-butyrolactone polymerisation on PEG macroinitiators via a crown ether-free anionic ring-opening polymerisation was reported providing good control on molar mass and molar-mass distribution of the final triblock copolymer [53,54].

**Figure 5:**
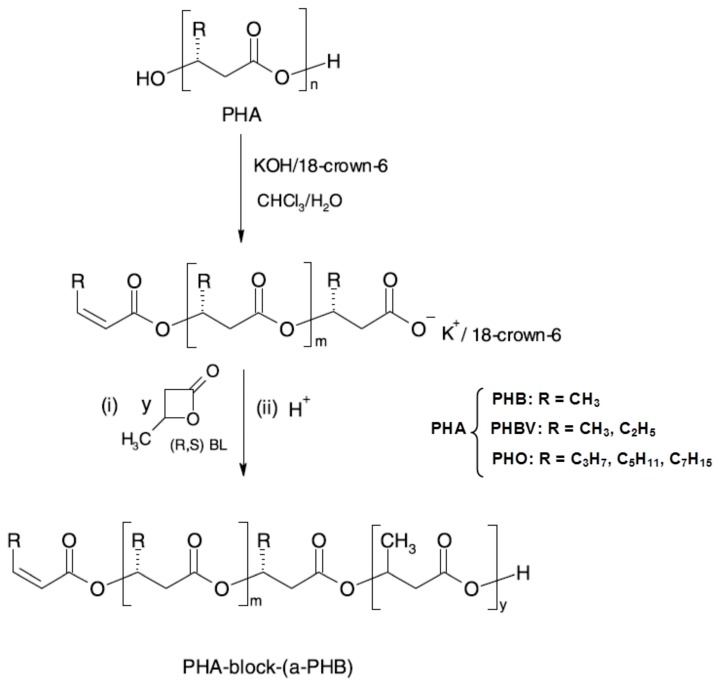
Preparation of block copolymers PHA-*block*-aPHB from low-molar mass macroinitiators, originally published in [61].

Block or graft copolymers may be also synthesised by means of macroinititors suitable for further specific chemical reactions [55]. Controlled depolymerisation of natural PHA catalysed by KOH/18-crown-6 complex in CHCl_3_/H_2_O system, due to the partial saponification of ester linkages followed by elimination reaction, leads to the formation of macroinitiator possessing unimodal molar-mass distribution and containing carboxylate end groups with K^+^/18-crown-6 counterion [56,57]. Such oligomers have been used for the characterisation of several PHAs at the molecular level, and their sequence distribution has been established by ESI-MS^n^ fragmentation experiments [57,58,59,60]. The low-molar mass macroinitiators of natural poly(3-hydroxyalkanoate) containing olefinic and active 18-crown-6 ether carboxylic end groups, are suitable to initiate ring-opening polymerisation of racemic β-butyrolactone. By this method, new block copolymers were obtained which combine selected natural PHA with poly[(*R*,*S*)-3-hydroxybutyrate)] (Figure 5) [61].

The physical properties of the obtained block copolymers, characterised by ^1^H-NMR, GPC and DSC showed that the composition and sequence distribution of the resulting oligomers was the same as in the starting material. Selected copolymers were subjected to hydrolytic degradation studies. The possibility to apply the obtained PHO-*block*-aPHB oligomers (PHO—poly[(*R*)-3-hydroxyoctanoate]) as compatibiliser for PHO/aPHB blend was confirmed. Moreover, the suitability of obtained polymeric materials for cardiovascular engineering was positively verified. The studies of permeability (performed according Polish norm PN-79/P04884.03) showed that vascular prosthesis covered with PHB-*block*-aPHB copolymer was the more watertight as compared with pure prosthesis [61].

Generally, use of poly[(*R*,*S*)-3-hydroxybutyrate], which is atactic and amorphous synthetic analogues of natural PHB, reduces the crystallinity of the copolymer or blend systems [62,63].

### 3.4. Utility and (Bio)degradation of Poly(3-hydroxyalkanoate)s

**Figure 6:**
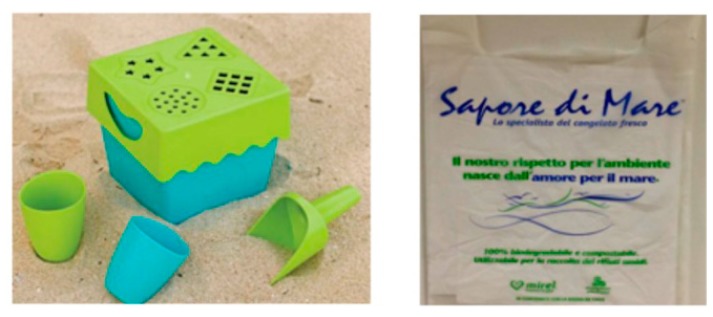
Zoe B Beach Toys, Compostable Shopping Bag, based on Metabolix, Inc. [67].

Processing properties of natural poly[(*R*)-3-hydroxybutyrate] are similar to those of petroleum-based polypropylene (PP.) [64]. VINNEX^®^, poly(vinyl acetate) based resins, enhances the physical properties of the material containing PHB, which significantly simplifies the processing. This, combined with the high heat resistance of PHB, allows the possible use of this material in applications such as hot filling [65]. Many practical applications in medicine, agriculture and in the food industry take advantage of the biodegradability of the PHAs, especially PHB and its copolymers with (*R*)-3-hydroxyvalerate (PHBV, Biopol^®^, Zeneca BioProducts) or with (*R*)-3-hydroxyhexanate (PHBH, Nodax™, P&G-Kaneka). Metabolix, Inc., an innovation-driven bioscience and engineering company, is developing and commercialising a family of high-performance PHA biopolymers targeted to the markets for performance additives, including film, toys, bags, *etc.* (Figure 6) [66].

In addition to the PHA producers from the U.S. (Metabolix Inc., Cambridge, MA, USA; Meredian Inc., Bainbridge, GA, USA) in Europe Biomer (Krailling, Germany) produces PHA under trade name of the Biomer^®^ [68]. PHAs are also produced in Brazil by PHB Industrial SA (Sao Paulo, Brazil), in the amount of about 50 tons of PHB and PHBV per year from sugar cane. In recent times, new Chinese companies appeared on the PHA market such as Tianan Biologic Material Co., Ltd. (Ningbo, China) producing PHBV and Tianjin Green Bio-Science Co., Ltd. (Tianjin, China), which produces in collaboration with DSM Venturing 10,000 tons of poly(3-hydroxybutyrate-*co*-4-hydroxybutyrate) per year [69,70,71]. Selective use of biodegradable materials in certain applications may provide a solution to the above-mentioned environmental problems. Goods from PHA polyesters applied as green biodegradable packaging involve foils, bottles and containers [72]. The presented results on biodegradation PHBV showed that water is a prerequisite for enhanced microbial growth and the enzyme-substrate contact. The PHA was also found to biodegrade in the tropical climate and marine environment [73,74,75,76,77]. The degradation of aliphatic polyesters to common metabolites in a biological environment, including anaerobic and aerobic conditions, results from enzymatic attack or simple hydrolysis and depends on different abiotic parameters of degradation environment [78,79].

Synthetic poly[(*R*,*S*)-3-hydroxybutyrate] has been paid much attention as a suitable modifier in blends with such (bio)degradable polymers as brittle polylactide or highly crystalline natural poly[(*R*)-3-hydroxybutyrate]. Earlier studies demonstrated that selected crystalline polymers (natural PHAs, polypivalolactone, polycaprolactone, poly(l-lactide)) after blending with aPHB successfully underwent enzymatic hydrolysis [62,63,80,81,82,83]. Moreover, the hydrolytic degradation of aPHB, natural PHBV and thereof blends preferentially occurs in the amorphous state by random scission of polyester chains. The increase in the content of crystalline phase derived from PHBV significantly reduces the rate of hydrolytic degradation of the blends tested. Thus, the hydrolytic degradation rate also was dependent on degrees of crystallinity of the polymers. Synthetic amorphous PHB shows the fastest rate of hydrolytic degradation. Bacterial PHB, which has the highest crystallinity degree, degrades most slowly, while PHBV having a lower crystallinity degree than bacterial PHB has an intermediate rate of degradation [81]. In order to improve the mechanical or chemical properties and to reduce the price of the polymeric materials, while maintaining their biodegradability the composites of natural polymers and fillers are developed. The biodegradable composites very often contain natural fibres (e.g., hemp) or sawdust [84,85,86,87]. Previous results indicate that the natural PHBV and its binary blends containing aPHB degrade enzymatically in compost with activated sludge and in the dynamic Baltic Sea water. The degradation degree of samples incubated in compost is higher than those of the samples incubated in seawater. Moreover, the degradation degree of the blends was dependent on the aPHB content [88]. Degradation of aPHB binary blends with natural PHB and poly(l-lactide), respectively, has been also investigated in soil. The increase in the number of microorganisms observed particularly for the soil where binary blends were incubated, indicating the microbial degradation of aPHB. The terrestrial plant growth test (cress and barley) demonstrated no toxicity of the materials studied on the environment [89]. Recently, the PCL blends as food packaging with excellent durability and mechanical properties have been studied [90]. The synthesis and toxicity studies of well-defined oligo[(*R*,*S*)-3-hydroxybutyrate]s and their potential application as drug delivery carrier systems were also presented [91]. The chemical structure of OHBs is similar to that of high-molar-mass natural PHB, it is pure and does not contain toxic impurities and it can be useful for drug modification [40,92]. Furthermore, an anti-proliferative activity of ibuprofen-OHB conjugates against colorectal cancer cells *in vitro* was also demonstrated [93].

When biodegradability of a given material is combined with low toxicity, a biotechnological product of great commercial interest could be achieved. Biomedical applications of PHAs, such as biodegradable controlled antibiotic release systems, implants, sutures and other medical devices offer the highest growth during the last years. In medicine PHAs, in pure form or as composites with other materials, applications have been found as implants for targeted drug delivery and scaffolds as well as in tissue engineering. Oligohydroxyalkanoates can also be used as a calcium influx stimulant for drug applications [24,94].

In the case of low purity PHA, obtained from activated sludge or nutrient-rich wastewater, it could find application in the energy industry. Production of PHA by mixed-culture composition in the open system can reduce the production costs of PHA for biofuel applications [95]. Acid, alkali or enzyme-catalysed hydrolysis of PHAs as well as biotransformation or fermentation of appropriate strains leads to the corresponding methyl esters of hydroxyalkanoates. These compounds are good fuels, alone or as additives to other fuels. PHA biofuels possess high oxygen content and while not containing nitrogen and sulphur. Besides, PHA biofuels could be a good alternative for the traditional biofuels such as bioethanol or biomethanol which was produced from biomass [29,96].

According to Markets and Markets, a global market research and consulting company based in the United States, PHA market consumption will grow from an estimated 10,000 million tonnes in 2013 to 34,000 million tonnes by 2018. PHAs are currently used in food service, biomedical and agricultural applications, electronic, packaging, automotive and chemical industries, as well as in photographic applications and the printing industry. The largest area of PHA use is packaging followed by food services. The increasing demand for renewable and bio-based materials and shift in consumer preference for eco-friendly products is driving the global market of polyhydroxyalkanoate. The PHA market has only few companies but it has been making rapid technological advancement along with increased investments in R&D. Therefore, PHA will still be commercialised and used in a wide range such as packaging, food service, biomedical and agriculture [71].

## 4. Polylactide—Synthetic Polymer from Renewable and Synthetic Monomers

Polylactide (PLA), an aliphatic degradable and biocompatible thermoplastic polyester is polymer with a high potential particularly for medical and packaging applications [97,98]. One of its most promising applications is its use for the production of (bio)degradable and biocompatible materials as an environmentally friendly alternative to non-biodegradable plastics derived from petrochemicals [99,100].

### 4.1. Synthesis of Polylactide and Polylactide-Based Copolymers, Blends and Composites

PLA can be obtained from natural or synthetic monomers. To produce natural monomer from renewable resources, biomass, in particular starch, cellulose and lignin, is converted into lactic acid (mostly l-isomer) in a fermentation process of carbohydrates (maltose, sucrose, lactose, *etc.*) and then chemically transformed to polymer [16,101]. Polylactide can be obtained by several methods. The conventional polycondensation of lactic acid does not increase sufficiently the molar mass of the resulting PLA due to water formation that negatively influences esterification equilibrium. Therefore, the most common method to obtain high-molar-mass polylactide is through ring-opening polymerisation of the cyclic lactide dimer [102,103,104,105,106].

Direct polycondensation is performed either in solution or in melt. It is possible to obtain PLA with a molar mass of 100,000 g/mol in one step polycondensation of lactic acid in solution using high-boiling-point solvents and molecular sieves as drying agents for the effective removal of water. It was also found that the tin oxide and chloride can effectively increase the PLA molar mass during melt condensation [107].

The ring-opening polymerisation requires the use of heavy-metal-based catalysts, such as zinc and stannous oxides, zinc and tin chlorides or stannous octoate, which often contaminate the resulting polymer. Therefore, polymerisation of lactide using zirconium(IV) acetylacetonate [Zr(acac)_4_] as an initiator was investigated for medical applications [108]. The enzymatic synthesis is also considered an environmentally friendly method, when carried out under mild conditions and can provide adequate control of the polymerisation process [109]. Table 1 shows some of the catalysts commonly used for the synthesis of polylactide.

The copolymerisation of lactide with other lactone-type monomers can improve properties such as high crystallinity, high melting point, and poor solubility [107]. PLA copolymers with PCL and/or polyglycolide (PGA) were synthesised by ring-opening polymerisation of corresponding cyclic lactones, *i.e.*, lactide, ε-caprolactone, or glycolide via anionic, cationic, coordination-insertion or enzymatic mechanism [6,110,111]. The synthesis of PGA/PCL copolymers were conducted using different conditions which lead to various chain microstructures and resulted in different degradation rate. The segmental microstructure of the copolymers chain which consists of the sequences containing longer lactidyl microblocks (GGGGG, LLLLL, GLGGG, GGGLG, LGGLG or GLGLG) and short LGL-segment, which originate from intermolecular transesterification were obtained during copolymerisation initiated with Zr(acac)_4_ conducted in bulk at 110 °C [112,113,114]. Furthermore, PLGA can be synthesised by direct melt polymerisation of the hydroxy acids, lactic and glycolic acid. The copolymer poly(lactide-*co*-glycolide) is one of the most interesting polymers for medical applications. This bioresorbable, biocompatible and non-toxic polymer has also good degradability which can be modified by different copolymerisation ratio of the monomers [115]. Copolymers of l-lactide with 25%–70% glycolide are amorphous. There is no linear relationship between the copolymer composition and the mechanical and degradation properties of the copolymers. The rate of degradation depends not only on the LA/GA ratio but also on the molar mass and the shape and structure of the polymer matrix [18,19]. The poly(lactide-*co*-glycolide) are in glassy state during degradation process because their glass-transition temperature is higher than 37 °C, which accounts for the low water absorption and slow drug release [116]. To modify the biological and mechanical properties of PLGA composites with carbon fibres and hydroxyapatite was prepared [117,118,119,120].

**Table 1:** The catalysts used for the preparation of PLA based on [102].

Polylactide *	Catalyst	Solvent	Molar Mass
PDLLA/PLLA	Aluminium isopropoxide	Toluene	*M*_n_ = 90,000
PDLLA	Stannous octoate	Alcohols	*M*_W_ < 350,000
PLLA	Stannous octoate	Alcohols, carboxylic acids	*M*_n_ = 250,000
PLLA	Stannous octoate, titanium or zirconium compounds	Toluene	*M*_n_ = 40,000–100,000
PDLA/PLLA/PDLLA	Stannous trifluoromethane sulfonate, scandium(III) trifluoromethane sulfonate	Ethanol	–
PLLA	alkoxides of Mg, Al, Zn, Ti	Methylene chloride	–
PLLA	Yttrium tris(2,6-di-tert butyl phenolate) in toluene	2-Propanol, butanol, ethanol	*M*_n_ < 25,000
PDLLA	Zn lactate	Bulk	*M*_n_ = 212,000
PDLLA/PLLA	Butylmagnesium halides (Grignard reagents)	Ethers	*M*_n_ < 300,000
PLLA	Potassium naphthalenide	THF, toluene	*M*_n_ < 16,000
PLLA	Complexes of iron with acetic, butyric, isobutyric or dichloroacetic acid	Bulk	*M*_W_ = 150,000

* PLLA—poly(l-lactide); PDLA—poly(d-lactide); PDLLA—poly(d,l-lactide).

### 4.2. Properties, Utility and (Bio)degradation of Polylactide

Polylactide has attracted much attention because of its excellent material properties which allow a wide range of potential applications in many fields as medical devices, textile, packaging applications and in sustainable development, recycling as well as environmental protection [98]. Polylactide can be a semicrystalline or fully amorphous material, depending on the stereochemical structure and thermal history, and may exhibit crystal polymorphism [6,121,122]. The *T_g_* of polylactide ranges from 50–80 °C, and the *T_m_* ranges from 130–180 °C [6,123]. The polymer architecture, crystallinity and molar mass are the most important parameters that affect the mechanical, chemical, and thermal properties of the polylactide materials [6,124]. PLA is suitable for large-scale production of materials with good mechanical and high barrier properties, resistance to fat action and UV rays, which are close to those of conventional oil-based polymers [6,125,126]. Although PLA is sensitive to processing conditions, it can be processed by injection molding, hot press molding, spinning, blow molding, foam molding or electrospinning [6,127]. Under extrusion, rapid decrease in viscosity and molar mass is observed due to macromolecular chain cleavage induced by increased shear level, temperature, and/or residence time [6,128]. Poly(l-lactide) is a highly crystalline (of about 37%), isotactic polymer with good tensile strength, low extension, high modulus (approximately 4.8 GPa), higher *T_g_* and slower degradation time compare to the amorphous form. It is preferred in applications where higher mechanical strength and toughness are required [18]. On the other hand, poly(d,l-lactide) (PDLLA) is an amorphous polymer having a random distribution of both isomeric forms of lactic acid [6]. The degradation product of PLA, lactic acid, a normal human metabolic by-product is converted into water and carbon dioxide via the citric acid cycle [18].

Polylactide is widely considered to be a biodegradable polymer, however, poly(α-hydroxy acid)-type polyesters are now being proven to degrade *via* simple chemical hydrolysis or enzymatically, despite the fact that some exotic enzymes such as proteinase K can cleave their main chain. Only oligomers (oligomeric degradation by-products at the last stages of the hydrolytic degradation processes) undergo enzymatic degradation but the role of hydrolysis *versus* enzymatic degradation is still under discussion [5,98,129]. Many studies have shown that PLA degrades completely in different environments [130,131,132,133,134,135,136,137,138,139]. Even the degradation of PLA films occurred in paraffin due to presence of the residual water content [140,141]. In the human body, the temperature of degradation is generally around 37 °C and PLA may be degraded *in vivo* in 2–3 years, while in the environment PLA can be degraded completely in a short time under composting conditions where the temperature can go up to 70 °C, as well as remains almost stable in water at a temperature of 20 °C [5]. The degradation of polylactide in aqueous medium occurs through hydrolysis (the random scission) of ester bonds autocatalysed by carboxylic acid end groups, and the hydrolysis rate increases with degradation time. Water absorption is a critical factor. For a time the partially degraded macromolecules remain insoluble in the surrounding aqueous medium and the degradation proceeds homogeneously according to autocatalysis rules. During the first step of hydrolytic degradation of PLA, the molar-mass dispersity increased and the molar mass decreased rapidly with a slight mass loss. In the next step, when the molar mass loss slowed down, the molar-mass dispersity decreased since low-molar-mass compounds were removed from the polymer matrix, and the catalytic effect of the carboxylic acid groups was reduced [5]. Subsequently, the molar mass loss slowed down with increasing mass loss. [142]. Faster internal degradation of PLA polymers is regarded as a general phenomenon. Degradation process, that combines diffusion, chemical reaction, and dissolution phenomena, results in a differentiation between the rates of degradation at the surface and interior of the matrix [5,143,144]. It is also known that submillimeter films degrade homogeneously and more slowly (erosion is restricted at the surface) than large-sized devices [145]. Moreover, porous systems generally degrade at a slower rate than plain ones because the release of soluble degradation by-products, that are responsible for the autocatalysis, can escape more easily from the polymer matrix as a result of the higher surface. It has been shown that structural differences affect the degradation characteristic in the case of stereocopolymers of PLA synthesised in the presence of different initiating systems [5].

Despite the complexity of PLA degradation mechanism, bioresorbable and biorecyclable lactic acid-based materials are presently at the commercial stages as matrixes to make surgical devices (wound closure in form of sutures, surgical clips, surgical staples and adhesives, tissue repair and regeneration scaffold as bone plates, screws and filling material for bone reconstruction or plastic surgery), controlled drug delivery particulate systems in pharmacology, and packaging [5,146].

For many applications, PLA could be blended with other polymeric or non-polymeric components in order to achieve the desired properties. Due to the low moisture absorption and high wicking, low flammability and smoke generation, UV-light resistance, relatively low specific gravity and low refraction index, and provision of excellent colouring characteristics, PLA is suitable for many applications such as outdoor applications and textile-fibre applications (shirts, furniture and automobile fabrics, carpets, wipes, table clothes, curtains, underwear and sportswear). In addition, disposable garments, awnings, feminine hygiene products, diapers production can utilise PLA. Foamed PLA may be used as structural protective material as well as in loose-fill packaging and insulation. PLA is also usable for coated paper, high-value films, rigid thermoformed containers, bottles, dog poop bags and a variety of other packaging applications [147,148,149].

Polylactide is used in agricultural applications such as mulching films, plant pots, compostable yard bags, bindings, clips, and other devices. Plastic agricultural mulches provide many benefits to the cultivation of specialty crops such as vegetables, including weed prevention and increased soil temperature, which leads to an increase in crop yield. Poly(lactic acid)-based mulches prepared using nonwovens textile technology are the perfect plastic mulch which would be ploughed into the soil at the end of the growing season, and undergo full mineralisation within a few months [147,150].

**Figure 7:**
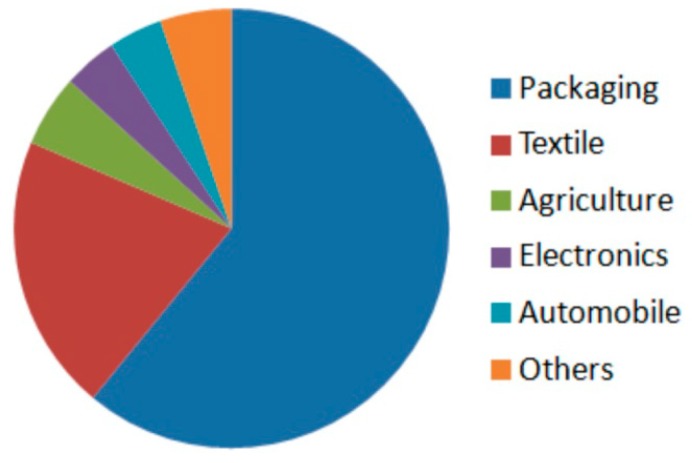
Polylactide market size in 2013 by application [154].

PLA was commercialised on a large scale particularly as a degradable, short-term packaging and agriculture applications and as a fibre (Figure 7). Cargill has developed processes that use corn and other feedstock to produce different PLA grades (NatureWorks LLC) [97,151]. Polylactide water bottles, commercially available from Biota, were obtained from NatureWorks LLC PLA (Blair, NE, USA). In NatureWorks LLC Company, the production of PLA in 2012 was estimated to be 140,000 tons. Currently, this is world’s largest production of biodegradable polyester with a price of less than 2 €/kg.

At present, the main European producers of PLA are Corbion Purac (Soesterberg, The Netherlands), Synbra Technology BV (Etten-Leur, The Netherlands), Pyramid (Guben, Germany) and Futerro (Escanaffles, Belgium, in 2007 joint venture between Galactic from Belgium and Total Petrochemicals from France) as well as US-based NatureWorks [97,101,152]. PLA consumption is around 200,000 tons per year. At present, only around 30% of lactic acid is used for PLA production [98]. However, the lactic acid market is projected to exceed $ 3577.5 million and poly(lactic acid) market to reach $ 4840.1 million by 2019, respectively [153].

## 5. Aliphatic Polyamides

### 5.1. Synthetic Pathways and General Properties of Aliphatic Polyamides

Aliphatic polyamides, also known as nylons (e.g., Nylon-66; Nylon-612; Nylon-46; Nylon-6; Nylon-12; *etc.*) are among the most important commodity polymers [155]. Polyamides are heterochain polymers containing amide groups in the macromolecular backbone. This large polymer encompasses thermoplastics of extremely broad range of available properties which are used in the production of films and fibres, moulding compounds, *etc.* [156,157].

Polyamides are generally synthesised by three methods: (i) polycondensation between diamines and dibasic acids; (ii) polycondensation of amino carboxylic acids as bifunctional monomers; and (iii) ring-opening polymerisation of lactams—cyclic amide monomers containing 3–7 ring atoms [158,159]. Synthetic pathways to polyamide copolymers of various structures and compositions are well covered in the literature [158,160,161,162,163].

The majority of polyamides are semicrystalline and generally very tough materials with good thermal and chemical resistance. The different nylon types give a wide range of properties and are used in many applications due to the combination of outstanding mechanical and electrical properties, particularly toughness and wear resistance (Table 2). Nylons also have excellent chemical resistance and can be used in high temperature environments. Heat stabilised reinforced systems allow sustained performance at temperatures up to 185 °C [164].

**Table 2:** Most important commercial polyamides [165,166,167,168,169].

Name and Chemical Structure	Melting Point (°C)	Typical Application	Producer (Trade Name)
Nylon-6 (PA 6) –[NH(CH_2_)_5_CO]_n_–	219–220	High-temperature applications; automotive industry; electrical and electronic industry (housings, plug and socket connectors, printed circuit boards); sports equipment.	BASF AG (Ultramid ^®^ B); DSM (Akulon^®^ and Novamid^®^); Honeywell Resins and Chemicals L.L.C. (Capron^®^); DuPont (Zytel^®^); Rhodia (Technyl^®^).
Nylon-66 (PA 66) –[NH(CH_2_)-NHCO-(CH_2_)_4_CO]_n_–	260–300	Industrial yarns and textile; automotive industry (radial tires; intake manifolds, engine covers, gears).	DuPont (Zytel^®^); BASF AG (Ultramid^®^ A); DSM (Akulon^®^ S); Rhodia (Technyl^®^); Ascent Performance Materials (Vydyne^®^).
Nylon-610 (PA 610) –[NH(CH_2_)_6_-NHCO-(CH_2_)_8_CO]_n_–	211–236	Engineering and construction materials; industrial parts, tubings, rods and profiles; sheet applications.	BASF AG (Ultramid ^®^ S); Rhodia (Special Technyl^®^ grades); Toray Resin Company (Amilan^®^).
Nylon-612 (PA 612) –[NH(CH_3_)_6_-NHCO-(CH_2_)_10_CO]_n_–	206–246	Pipes, bushings, electrical connectors, industrial parts, bristles, tubings, rods.	DuPont (Zytel^®^ 150 series); Evonik (Vestamid^®^ D series).

Nowadays, more attention is focused on the attractive class of bio-based polyamide thermoplastics, which are partly or wholly made from renewable resources [170,171]. In the production of such polyamides, bio-based monomers derived from castor oil or mass produced by fermentation are applied. Synthetic pathways to obtain bio-based polyamides are basically the same as to synthetic polyamides and there are a number of commercial products available on the market (Rilsan^®^11 of Arkema (Colombes, France); Ultramid Balance^®^ of BASF (Ludwigshafen, Germany); Vestamid Terra^®^ of Evonik (Essen, Germany); *etc.*) [172].

Poly(ester-amide)s are another fast developing family of thermoplastics that combine the valuable properties of both polyesters and polyamides, *i.e.*, polyester’s biodegradability and polyamide’s high thermal stability and high tensile strength [173]. Particular attention is focused on the poly(ester amide)s built of α-amino acids which became important materials in the biomedical field [174]. The presence of the α-amino acid contributes to better polymer-cell interactions and allows the introduction of functional groups thus enhancing the overall biodegradability of the material.

### 5.2. Utility and Biodegradation of Aliphatic Polyamides

Nylons are available for processing *via* injection moulding, rotational moulding, casting or extrusion into film or fibre. For industrial uses, polyamides persistently replace traditional materials in applications ranging from showpiece examples such as artificial organs and construction materials for moon-based space stations through to the more routine but equally important uses, including high load bearings, wear pads, support and guide wheels, buffer pads and gears, and many more [175].

**Figure 8:**
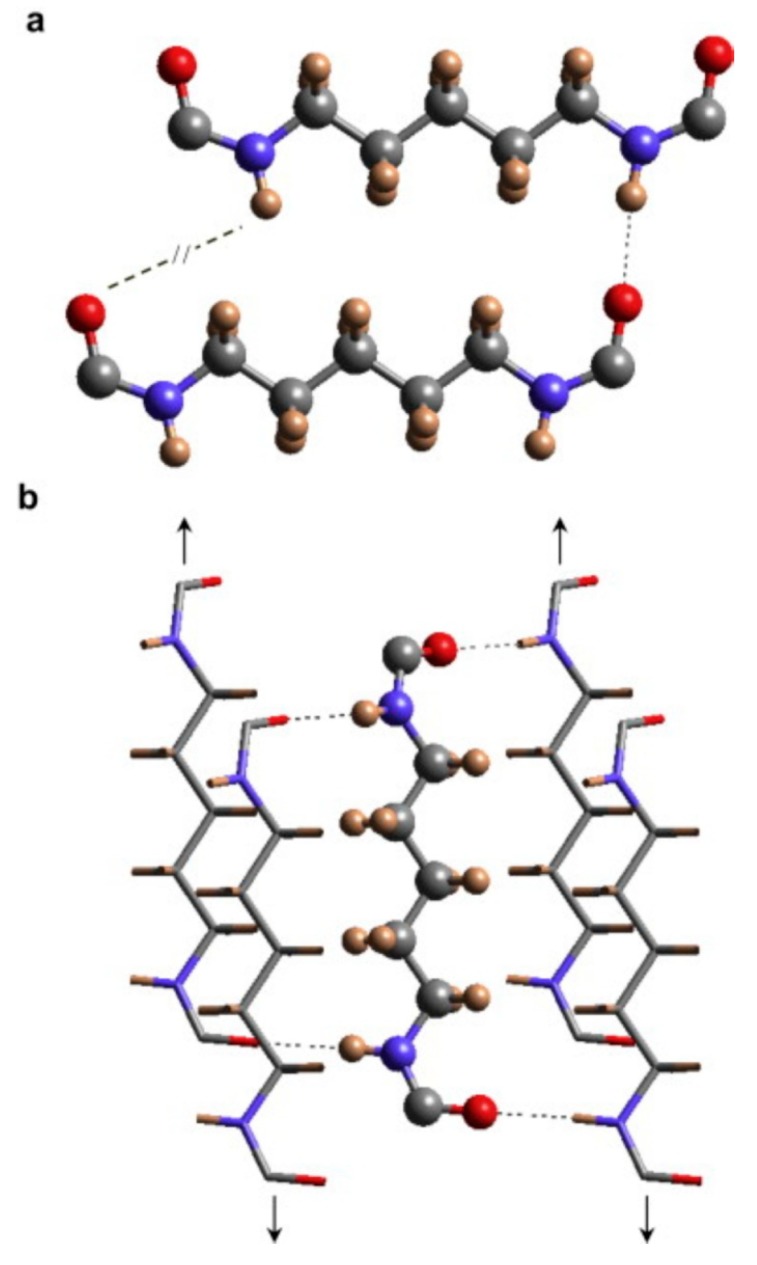
Establishment of hydrogen bonds between Nylon-56 molecular chains, (**a**) scheme of the unfavourable hydrogen-bond geometry between odd diamide units of Nylon-56 molecular chains with an all trans conformation and (**b**) scheme of the establishment of hydrogen bonds along two directions when consecutive amide planes of a molecular chain slightly rotate in opposite directions from the plane defined by the methylene carbon atoms (nitrogen, blue; oxygen, red; carbon, gray; hydrogen, brown), originally published in [180].

Biodegradation of synthetic polyamides is generally known to be poor, although its chemical structure (presence of amide bonds in the main chain) resembles those of natural proteins and synthetic polypeptides [9,176,177]. The high resistance to degradation of synthetic polyamides is caused mainly by the high symmetry of their molecular structures and strong intermolecular cohesive force caused by hydrogen bonds between molecular chains, which results in highly crystalline morphology (Figure 8) [178,179,180]. However, several papers and reviews report on the degradation of oligomers and higher molar mass polyamides by different microorganisms and enzymes [178,181]. Nylon-66 and Nylon-6 are significantly degraded by enzymes from white-rot fungus *Bjerkandera adusta* [182,183,184]. Biodegradation of Nylon-6 oligomers is also observed with *Pseudomonas* and *Flavobacterium* [178]. Andreoni *et al.* [185] described three mixed cultures of aerobic bacteria able to grow on 6-aminocaproic acid polyamides of low molar mass (up to 6800 g/mol), but biodegradability of polyamides of molar mass higher than 11,000 g/mol was not reported. The extent of the bacterial growth was found to dependent on the amount of cyclic and linear oligomers present in the polymeric matrix.

Although made of naturally occurring substances, bio-based polyamides are not necessarily readily biodegradable. Nevertheless, bio-based Nylon-4 and some end-group modified analogues are easily biodegraded by bacterium *Pseudomonas* sp. strain ND-11 isolated from activated sludge. It is shown that polyamide degradation in this artificial environment proceeds via hydrolysis [186,187]. Biodegradability of Nylon-4 in composted soil is also reported. [188,189,190]. In addition, Tachibana *et al.* [191] investigated the biodegradability of Nylon-4 films in seawater from the Tokyo Bay. The slower plastic degradation in marine environment is due to the lower temperature and plastics degradation into smaller pieces that persist into the seawater [192].

Nylon-12 and Nylon-66 could also biodegrade by fungi and bacteria. Deguchi *et al.* reported that the white rot fungi strain IZU-154, a kind of lignin-degrading microorganisms, degraded Nylon-66 films through oxidative processes [182,193]. *Geobacillus thermocatenulatus* could also provide Nylon-12 and Nylon-66 biodegradation [194]. Bacterial degradation of Nylon-12 is usually associated with the enzymatic hydrolysis of amine bonds, which is accompanied by the formation of 12-amino dodecanoic acid. Tomita *et al.* [195] reported on a thermophilic strain isolated from 100 soil samples by enrichment culture technique at 60 °C which is capable of degrading Nylon-12. At this temperature, the strain grew on Nylon-12, accompanied by a marked decrease in molar mass of Nylon-12. The strain is also capable to degrade Nylon-6 as well as Nylon-12, but not Nylon-66.

Although Nylon-6 is generally regarded as a non-biodegradable polymer [196], the aforementioned fungi strain IZU-154 could also degrade Nylon-6 fibres to soluble oligomers [182]. Freidrich *et al.* [170] reported on the screening of a large number of fungi for their ability to degrade high molar mass Nylon-6. The study showed that the white rot *Basidiomycetes* is able to degrade Nylon-6 when grown on this polyamide as the only N-source. For the first time, this polymer was shown to be disrupted by *Bjerkandera adusta.* The remaining insoluble part of the nylon showed a decrease in number-average molar mass from 16,900–5600 g/mol during 60-day incubation. It was assumed that the insoluble polymer was partially solubilised and metabolised by the fungus.

Biodegradation of high molar mass polyamides could be increased by the introduction of various side groups as hydroxyl, methyl and benzyl or through copolymerisation [197]. In this context, poly(ester-amides)s are probably one of the most promising biodegradable structures among the various amide copolymers. General review on the synthesis, properties and degradation (although mostly enzymatic) of poly(ester-amide)s is given in many publications [173,174,198]. The increasing interest in poly(ester-amide)s as a biodegradable materials for environmental applications prompts a thorough study of the biodegradation process in nature, degradation products and its biocompatibility, *etc.* For example, Okada *et al.* [199] reported on biodegradation of eight different poly(ester amide)s obtained by solution polycondensation of the *p*-toluenesulfonic acid salts of *O*,*O'*-bis(α-aminoacyl)-1,4:3,6-dianhydro-d-glucitol and bis(*p*-nitrophenyl) esters of aliphatic dicarboxylic acids. The poly(ester-amide)s obtained ranged from amorphous to semicrystalline, and were all soluble in various polar solvents. Soil burial degradation tests of the films of investigated poly(ester amide)s, followed by SEM observation, indicated that all structures are biodegradable. Moreover, their biodegradability was dependent on the molecular structure. Comparison of the biodegradability of the poly(ester amide)s with that of the corresponding polyesters showed that the poly(ester amide)s were, in general, less readily degraded in composted soil than the corresponding polyesters having the same aliphatic dicarboxylic acid unit.

## 6. Conclusions

Currently, when the environmentally safe and sustainable development is crucial to the society research, production, and utilisation of (bio)degradable polymers becomes an increasingly important issue. In view of fact that (bio)degradable polymers derived from either renewable or fossil recourses attract increasing attention due to their immense impact in everyday life, (bio)degradation should be a key feature of these materials in order not to accumulate in the environment and reduce the ecological risk. The environmental impact of polyester-based (bio)degradable polymers is still under discussion. The most important advantages and limitations from our point of view are summarised in Table 3 [200,201,202].

**Table 3:** The environmental impact of polyester-based (bio)degradable polymers.

Polymer	The Impact on Sustainable Environment	
	Advantages	Limitations
PHA	Polymer from renewable natural resources; produced by bacteria as storage material; commercial production by living organisms using biochemical processes; biodegradable polymers with lack of toxicity, converted to the same metabolites as in living organisms: water and carbon dioxide; reduction of fossil energy (“old carbon”) usage.	Availability of waste collection systems and recycling methods; compostable plastic waste deposited on a landfill has a negative social environmental impact; approval of new bioplastics by society requires high level of customers’ awareness which depends on capital and education expenditure; too energy-intensive extraction stage.
aPHB	Synthetic polymer biodegrade under appropriate conditions (in the presence of PhaZ7 depolymerase from *Paucimonas lemoignei*) to form of monomer, dimer and trimer.	End-of-life treatment problems; post-synthetic residues (e.g., organic solvents).
PLA	Synthesis from renewable monomer; easily hydrolytically degradable polymer; less greenhouse gases emission and less consumption of non-renewable energy than traditional polymers.	Availability of waste collection systems and recycling methods; compostable plastic waste deposited on a landfill has a negative social environmental impact; approval of new bio-based plastics by society requires high level of customers’ awareness which depends on capital and education expenditure.
Polyamides	Possible synthesis from renewable monomers. Bio-based polyamide and poly(ester-amide)s thermoplastics of valuable properties.	Biodegradation possible only for polyamides of low molar mass; environmental impact still under evaluation.

In addition to the biodegradable polymers which are converted to carbon dioxide and water under appropriate conditions, there is an emerging class of oxo-degradable polymers, often incorrectly referred to as oxo-biodegradable polymers although they are only disintegrated, but not mineralised. Therefore, the various stages of biodegradation (biodeterioration, biofragmentation and assimilation) need to be precisely investigated and validated [6,203].

(Bio)degradation research on aliphatic polyesters, which are currently among the most promising groups of (bio)degradable polymers, is mainly focused on the degradation in soil or compost. However, aqueous environments are also very important, since they are the major routes of entry of polymeric packaging materials into the environment. For this reason, studies on the biodegradation pathways and conditions under which the biodegradation may occur when comparing different environmental compartments could considerably improve the development of (bio)degradable polymeric materials. It is also very important to ensure a real biodegradability and incorporation into geochemical life cycle rather than to provide materials that can disintegrated only to small particles [203].
